# 用于离子色谱系统淋洗液中CO_2_脱除的气液分离器设计与应用

**DOI:** 10.3724/SP.J.1123.2024.03021

**Published:** 2025-04-08

**Authors:** Yonghan PENG, Jingwen TANG, Yihua LI, Feifang ZHANG, Bingcheng YANG

**Affiliations:** 华东理工大学药学院,上海 200237; School of Pharmacy, East China University of Science and Technology, Shanghai 200237, China

**Keywords:** 离子色谱, 气液分离器, CO_2_脱除, 阴离子分析, 抑制器, ion chromatography (IC), gas-liquid separator (GLS), carbon dioxide removal, anion analysis, suppressor

## Abstract

设计了一种可用于脱除离子色谱(IC)系统淋洗液中CO_2_的气液分离器(GLS)。首先将微孔中空纤维聚丙烯管(PP-T)内插到内径适配的聚四氟乙烯管(PTFE-T)中,形成双套管结构并缠绕成螺旋形;随后将PP-T和PTFE-T的两端分别固定于配备了三通接头的两个水平接口处,将再生液管路固定在配备了三通接头的垂直臂接口处。当碳酸根淋洗液从上游抑制器流入PP-T内部时,经抑制作用后的碳酸溶液会分解产生CO_2_气体,这些气体随后通过PP-T逸出,并进入位于PP-T与PTFE-T之间环状空间内的流动吸收液中,从而实现CO_2_的持续、有效脱除。实验优化了GLS的制作条件和操作条件,包括PP-T的长度、PTFE-T的内径、操作温度和吸收液的种类、浓度及流速等。实验结果表明,将40 mmol/L的KOH溶液作为吸收液时,在淋洗液流速为1 mL/min的情况下,CO_2_的脱除效率可达98%以上;较高的操作温度有助于提高CO_2_的脱除效率并降低基线噪声,本实验优化后的操作温度为40 ℃。将该GLS应用于碳酸根淋洗液的IC系统,在使用1.8 mmol/L K_2_CO_3_-3.2 mmol/L KHCO_3_(1∶1, v/v)混合淋洗液时,背景电导信号从未使用GLS的41.6 mV降低至使用GLS后的5.5 mV;以150 μmol/L常见阴离子(F^-^、Cl^-^、${{\mathrm{NO}}_{2}}^{-}$ 、Br^-^、${{\mathrm{NO}}_{3}}^{-}$ 、${{\mathrm{SO}}_{4}}^{2-}$ )混合标准溶液为标样,使用GLS后的信噪比(SNR)得到了明显提升(6.0~11.8倍)。此外,该GLS还可用于脱除氢氧根淋洗液体系下大体积样品中的CO_2_(${{\mathrm{CO}}_{3}}^{2-}$ 的峰面积下降了约80%),降低样品中${{\mathrm{CO}}_{3}}^{2-}$ 对其他阴离子分离产生的干扰。

## Abstract (en)

A gas-liquid separator (GLS) for the removal of carbon dioxide in the eluent of an ion chromatography (IC) system was developed. Firstly, the microporous hollow fiber polypropylene tube (PP-T) was inserted into the polytetrafluoroethylene tube (PTFE-T) with suitable inner diameter to form a double casing structure and wound into a spiral shape. Each terminal of the PP-T is 3 cm longer than that of the PTFE-T. Then the terminals of PP-T and PTFE-T were fixed respectively on two horizontal joints equipped with tee joints, and the regenerated liquid pipeline was fixed on a vertical arm interface equipped with tee joints. When the carbonate eluent flows into PP-T from the upstream suppressor, the inhibited carbonate solution will decompose and produce CO_2_, which then escapes through PP-T and enters the flowing absorption solution located in the annular space between PP-T and PTFE-T, so as to achieve continuous and effective CO_2_ removal. The production conditions and operating conditions of GLS were optimized, including the length of PP-T, the inner diameter of PTFE-T, the operating temperature, the type, concentration and flow rate of the absorption solution. The experimental results show that when 40 mmol/L potassium hydroxide solution is used as absorption solution, the removal efficiency of CO_2_ can reach more than 98% at the flow rate of 1 mL/min. Higher operating temperature is helpful to improve the removal efficiency of CO_2_ and reduce the baseline noise. The optimized operating temperature in this study was 40 ℃. When applying this GLS to the IC system for carbonate eluent, using a mixed eluent of 1.8 mmol/L potassium carbonate-3.2 mmol/L potassium bicarbonate (1∶1, v/v), the background conductance signal decreased from 41.6 mV without GLS to 5.5 mV after using GLS. Using a mixed standard solution of common anions (F^-^、Cl^-^、${{\mathrm{NO}}_{2}}^{-}$ 、Br^-^、${{\mathrm{NO}}_{3}}^{-}$ 、${{\mathrm{SO}}_{4}}^{2-}$ ) at 150 μmol/L as the reference sample, the signal-to-noise ratio (SNR) after applying the GLS has been significantly improved by a factor of 6.01 to 11.8 times. In addition, the GLS can also be used to remove CO_2_ from large volume samples in hydroxide eluent system, reducing the interference of ${{\mathrm{CO}}_{3}}^{2-}$ on the separation of other anions.

离子色谱(ion chromatography, IC)是目前最常用的无机阴离子分析技术之一。碳酸根淋洗液和氢氧根淋洗液是阴离子分析体系最常用的两种淋洗液。与氢氧根淋洗液相比,使用碳酸根淋洗液具有以下优势:(1)碳酸根淋洗液可通过高纯度的化学试剂直接配制得到;(2)空气中的CO_2_对碳酸根淋洗液产生的干扰较小;(3)在等度条件下将碳酸根淋洗液与碳酸氢盐溶液混合,易于实现分离选择性和分析时间的调控。然而,由于碳酸根淋洗液的抑制产物为碳酸,其背景电导比氢氧根淋洗液的抑制产物(水)高出40~60倍,这会导致目标物的检出限变高、线性范围变窄、系统峰受干扰程度大、梯度洗脱难度大等一系列问题。结合碳酸易分解成CO_2_和水的特性,若能够通过合适的方式去除CO_2_,则可以有效克服上述缺陷。基于此,Sundén等^[1]^最早尝试使用聚四氟乙烯管(PTFE-T)(1 mm i.d.×1.6 mm o.d., 2 m)作为气液分离器(gas-liquid separator, GLS),用于脱除淋洗液中的CO_2_,所得到的脱除效率约为90%,但该方法的缺陷是死体积大且峰展宽严重。Siemer等^[2]^将薄壁硅胶管(0.31 mm i.d. × 0.6 mm o.d., 3.6 m)浸没于0.1 mol/L的KOH溶液中,成功实现了约73%的CO_2_脱除效率。Shintani等^[3]^使用内部填充了12根纤维的聚丙烯管(PP-T)(0.4 mm i.d.×0.6 mm o.d., 3.6 m)作为GLS,成功实现了约85%的CO_2_脱除效率。在后续的研究中,Ullah等^[4]^改进了该GLS,使用壁厚为25 μm、内径为0.4 mm的PP-T,并在其外表面涂覆一层硅油以提高气液分离效率,通过优化操作温度等参数,最终实现了约99%的CO_2_脱除效率。然而,该方法的缺陷在于硅油的涂覆过程难以重现。除了使用不同材质的GLS脱气管,研究人员在吸收液选择方面也有不同的尝试。上述方法均使用水或碱性溶液作为CO_2_的吸收液,而此后又出现了利用真空法脱除CO_2_的新方式^[5]^。Masunaga等^[6]^报道了一种基于静态法的CO_2_脱除器,该脱除器使用氢氧根型阴离子交换树脂作为CO_2_的固体吸附剂,并将其和气体渗透性管路共同置于密封容器中;通过树脂对CO_2_的吸附作用,使容器内的气压降低,从而促进CO_2_的持续逸出和脱除。然而,阴离子交换树脂的容量有限,该CO_2_脱除器的长期、持续性运行能力有待进一步考察。

基于上述问题,本文将内径为0.2 mm的PP-T内置于内径适配的PTFE-T中,形成双套管结构,开发了一种用于脱除IC系统淋洗液中CO_2_的GLS。研究中直接将抑制器再生液作为CO_2_吸收液,简化了流路结构。通过优化GLS的制作条件和操作条件(包括PP-T的长度、PTFE-T的内径、操作温度和吸收液的种类、浓度及流速等),本文实现了较高的CO_2_脱除效率。本文设计的GLS可以有效降低碳酸根淋洗液的背景电导,提高信噪比(SNR),扩大碳酸根淋洗液的适用范围。

## 1 实验部分

### 1.1 仪器、试剂与材料

TOSOH离子色谱仪(日本东曹生物科技有限公司); EDG-100 KOH电致淋洗液发生器、AES-100电致膜抑制器和电导检测器均参照实验室前期工作^[7⇓-9]^自制而成。

K_2_CO_3_、KHCO_3_(纯度99%)购自北京百灵威科技有限公司;二氯乙酸(DCA,纯度99%)、三氯乙酸(TCA,纯度99%)以及KClO_3_、KF、KBr、KCl、KNO_2_、KNO_3_和K_2_SO_4_标准储备液(1000 mg/L)均购自美国AccuStandard公司。

PP-T购自明旺合成纤维厂;PTFE-T购自美国VICI公司;所有自来水样品均来源于所在实验室。除非另有说明,实验用到的溶液均由超纯水(电阻率18.2 MΩ·cm,默克密理博公司)制备。

### 1.2 GLS的构建

本文设计的GLS为双套管结构,内管为PP-T(0.2 mm i.d.×0.3 mm o.d., 2 m),外管为PTFE-T(0.6 mm i.d.×1.6 mm o.d., 1.94 m)。将PP-T和PTFE-T的两端分别固定于配有三通接头的两个水平接口,将再生液管路固定于配有三通接头的垂直臂接口,GLS的结构示意图见图1。碳酸根淋洗液从淋洗液进口(E-IN)进入后在PP-T中流动,随后从淋洗液出口(E-OUT)流出,进入下游电导检测器;CO_2_吸收液从再生液进口(R-IN)进入后,在PP-T与PTFE-T之间的环形区域内流动,再从废液出口(W-OUT)流出。在运行过程中,从抑制器流出的碳酸根淋洗液是经过抑制处理的溶液,其中碳酸根以碳酸分子的形式存在。

**图1:**
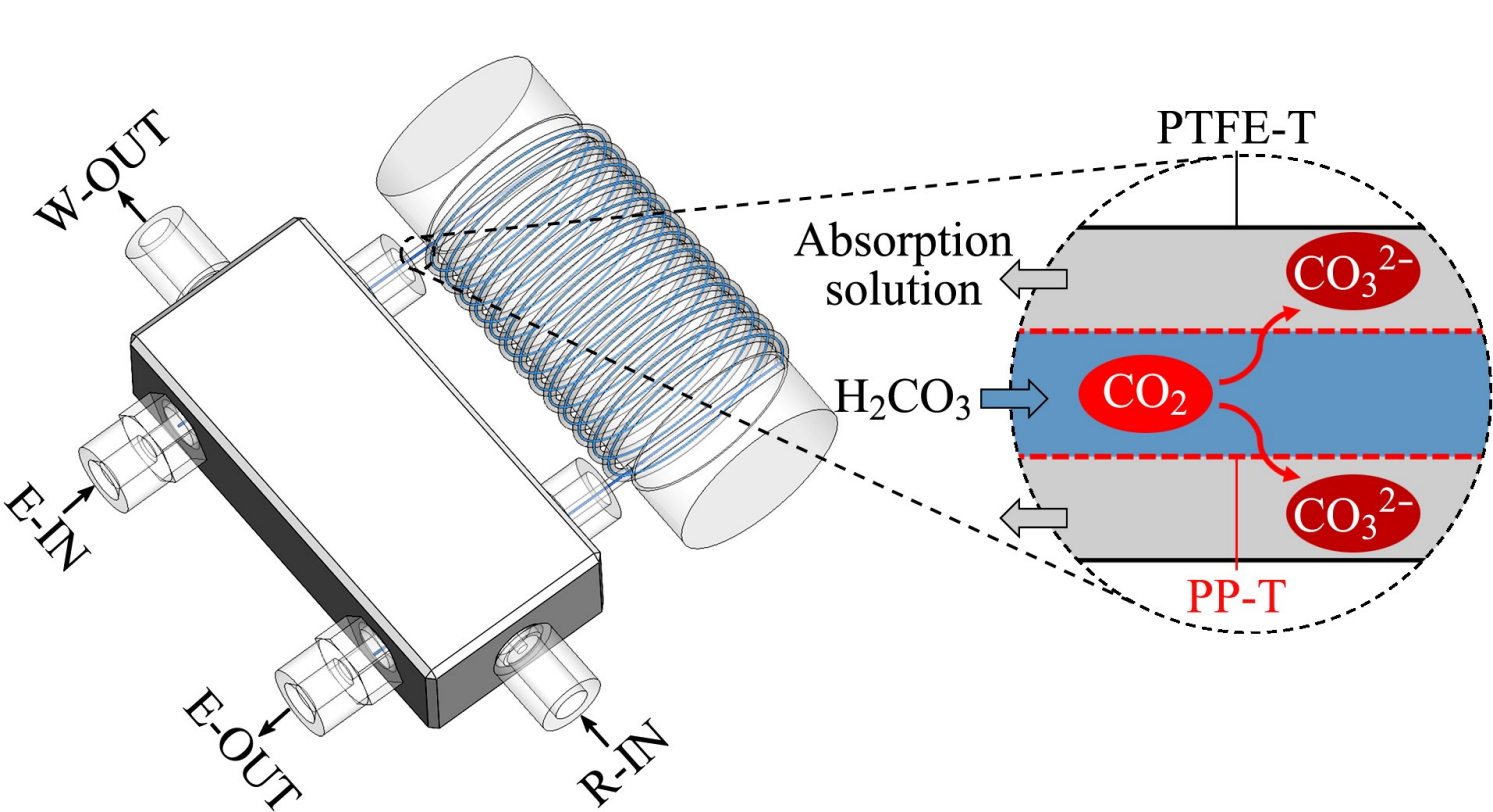
用于脱除CO_2_的GLS结构示意图

碳酸是一种弱酸,其在溶液中与CO_2_和H_2_O保持着动态平衡。PP-T内的CO_2_会因浓度梯度扩散至管外的碱性吸收液中,而吸收液对CO_2_的溶解和吸收又会进一步促进CO_2_的逸出,从而在宏观上表现为CO_2_的持续脱除过程。

### 1.3 实验条件

色谱柱:TSKgel SuperIC-AZ(150 mm×4.6 mm)、HS-5A-PS (250 mm×4.0 mm)、Thermo IonPac^TM^ AS18(250 mm×4.0 mm)。PP-T长度为2 m或3 m(根据具体实验情况而定); PTFE-T内径为0.6 mm;吸收液为由电致淋洗液发生器产生的KOH溶液,浓度为40 mmol/L,流速为1 mL/min;操作温度为40 ℃。

### 1.4 CO_2_脱除效率的计算

为计算GLS对CO_2_的脱除效率,首先需测定淋洗液中残留的碳酸浓度。本实验通过标定碳酸溶液浓度与背景电导之间的关系曲线来测定碳酸的浓度。由于碳酸溶液易分解,无法通过直接配制标准品得到,因此采取如下间接方案:通过泵将不同预设浓度的K_2_CO_3_溶液连续输送至电致膜抑制器,随后利用自制的非接触电导检测器^[9]^来产生电导信号。为抑制碳酸溶液发生分解,在非接触电导检测器后串联一个压力值约2 MPa的阻尼管。在经过抑制器充分抑制后,K_2_CO_3_会转化为等浓度的碳酸,随后根据电导信号拟合式(1)和式(2)来计算CO_2_的脱除效率(*E*,%):


(1) $$S=-0.667C_{1}^{2}+15.8C_{1}+6.41$$

(2) $$E=\frac{C_{2}-C_{1}}{C_{2}}\times 100\%$$

其中,*S*为电导信号(mV), *C*_1_为碳酸的浓度(mmol/L), *C*_2_为K_2_CO_3_的浓度(mmol/L)。

## 2 结果与讨论

### 2.1 GLS条件优化

#### 2.1.1 GLS制作条件的优化

当PP-T作为淋洗液通道时,其长度的增加会延长碳酸溶液在管内的停留时间,这有利于提升CO_2_的脱除效率并改善基线噪声。然而,长度的增加同时也意味着死体积的增大或柱外扩散现象的加剧,进而可能导致峰高降低或色谱分离度下降。因此,在设计中需要全面权衡这些因素。以50 μmol/L氯离子为标样,实验考察了PP-T长度(0、1.2、2、3 m)对背景电导、SNR及塔板数的影响,结果如图2所示。可以看出,当PP-T长度由1.2 m增加至3 m后,基线噪声和系统水负峰均明显下降,在3 m处降至最低(图2a);当PP-T长度由1.2 m增加至3 m后,SNR明显提高,但与此同时塔板数也会下降,并在2 m处降至最低(图2b)。因此,PP-T的长度(2 m或3 m)需根据具体的应用目标进行综合考量。将PP-T内置于PTFE-T中,在PP-T外径保持不变的条件下,PTFE-T的内径大小直接决定了吸收液的流动阻力,并进而影响CO_2_的脱除效率。实验考察了PTFE-T内径大小(0.6、0.75、1.0、1.6 mm)对CO_2_脱除效率及基线噪声的影响。结果表明,小内径的PTFE-T不仅有助于提升CO_2_的脱除效率,还能有效降低基线噪声;然而,进一步缩小内径会导致吸收液的动力学阻力增加,从而造成流动不畅。因此,本实验确定PTFE-T的内径为0.6 mm。

**图2:**
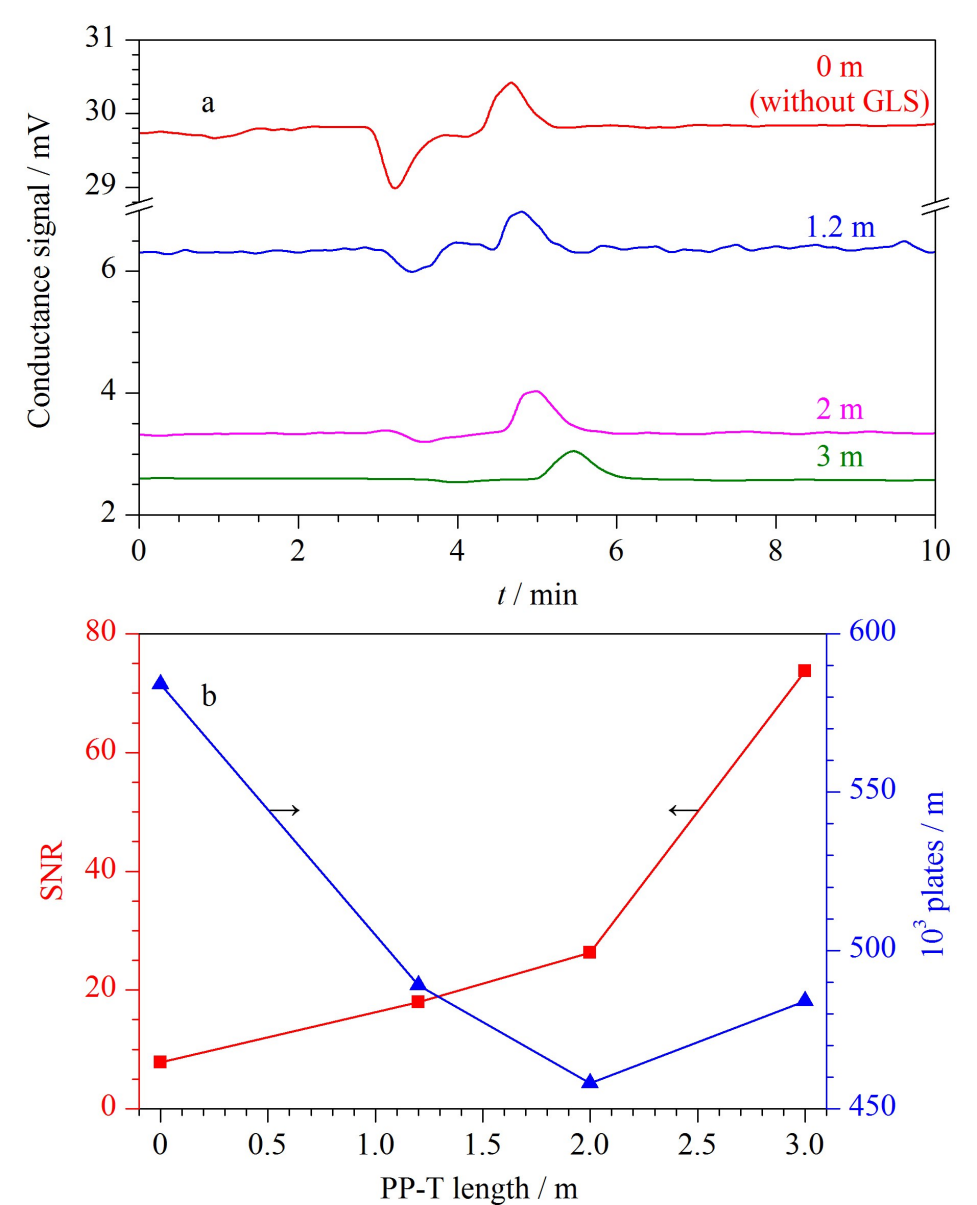
PP-T长度对(a)背景电导信号、(b)SNR及塔板数的影响

#### 2.1.2 吸收液种类、浓度及流速的优化

吸收液能够促进CO_2_的逸出,而不同种类的吸收液会对CO_2_的脱除效率及基线噪声产生影响。实验分别考察了纯水、20 mmol/L KOH溶液(通过电致淋洗液发生器产生)和来自电致膜抑制器的再生液(等效于20 mmol/L KOH溶液)3种吸收液对CO_2_脱除效率的影响。实验结果表明,在流速均为1 mL/min的条件下,使用3种吸收液所获得的背景电导分别为38.04、10.05和9.45 mV,噪声分别为0.167、0.079和0.153 mV, CO_2_脱除效率分别为78.0%、97.7%和98.0%。由于来自电致膜抑制器的再生液浓度无法随意调整,因此采用KOH溶液进行后续优化实验。

实验考察了不同浓度(20、40、60 mmol/L)的KOH溶液对CO_2_脱除效率的影响。结果表明,随着KOH溶液的浓度由20 mmol/L增至60 mmol/L, CO_2_的脱除效率也随之升高,但幅度较小。并且,当KOH溶液浓度≥40 mmol/L时,CO_2_已能够得到有效脱除(脱除效率≥98%)。鉴于在40 mmol/L的KOH浓度下基线噪声最低,故选择40 mmol/L KOH溶液用于后续实验。

随后,实验考察了吸收液(40 mmol/L KOH溶液)流速(0.2、0.4、0.6、0.8、1 mL/min)对CO_2_脱除效率的影响。实验结果表明,随着吸收液流速的增加,基线噪声呈现出先升高、后降低的趋势,且在1 mL/min处降至最低(图3)。随着吸收液流速的增加,CO_2_的脱除效率先增加,随后保持平稳。综合考虑,在1 mL/min的流速下,基线噪声降至最低点,同时CO_2_的脱除效率也能够达到98%以上。因此,最终确定吸收液的最佳流速为1 mL/min。

**图3:**
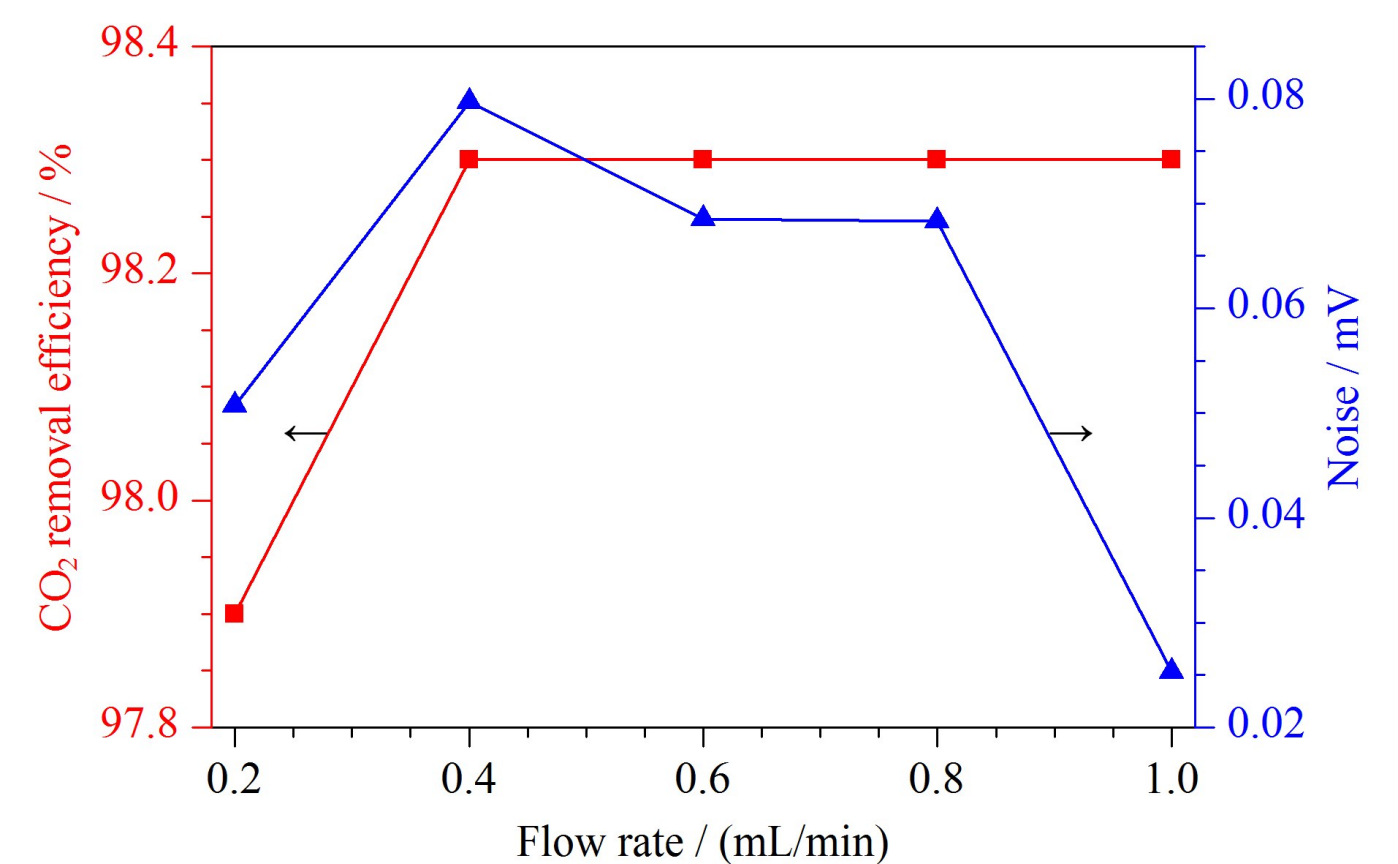
KOH溶液的流速对CO_2_脱除效率及噪声的影响

#### 2.1.3 操作温度的优化

在常压条件下,气体在溶液中的溶解度会随着溶液温度的升高而减小,因此提高操作温度将有助于提升CO_2_的脱除效率。实验考察了不同的操作温度(22、30和40 ℃)对系统噪声和CO_2_脱除效率的影响。结果表明,随着温度升高至40 ℃, CO_2_的脱除效率逐渐增加且基线噪声降低;然而,若进一步升高温度则不利于延长GLS的使用寿命。因此,选择40 ℃为最优操作温度。

### 2.2 GLS在IC系统中的应用

#### 2.2.1 GLS应用于碳酸根淋洗液的IC系统

碳酸根淋洗液与GLS高度兼容,实验以1.8 mmol/L K_2_CO_3_溶液-3.2 mmol/L KHCO_3_溶液(1∶1, v/v)为淋洗液,对比了GLS使用前后对系统背景电导信号的影响。结果如图4上图所示,在使用GLS后,背景电导由41.6 mV降低至5.5 mV;基线噪声由0.347 mV降低至0.129 mV。以150 μmol/L常见阴离子(F^-^、Cl^-^、N${O_{2}}^{-}$ 、Br^-^、N${O_{3}}^{-}$ 、S${O_{4}}^{2-}$ )混合标准溶液为标样,实验对比了使用GLS前后对阴离子分离效果的影响。结果如图4下图所示,在碳酸根淋洗液体系下,系统水负峰信号和基线噪声明显降低,背景电导也从20.8 mV下降至0.82 mV;另一方面,使用GLS后系统的死体积变大,导致柱外扩散现象加剧,部分样品峰高下降了1.8%~31.7%,而F^-^样品峰高上升了25%。

**图4:**
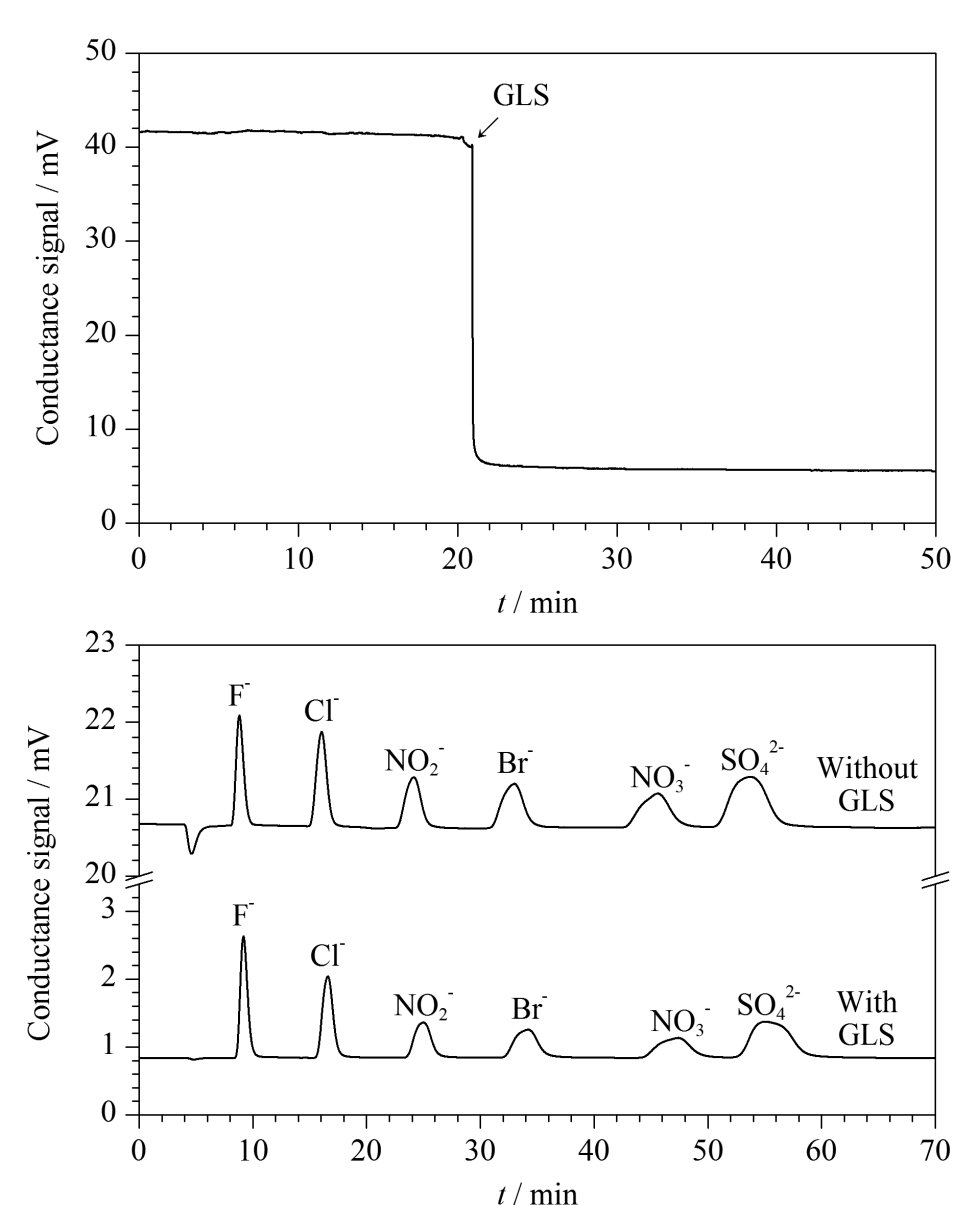
GLS应用于碳酸根淋洗液体系的效果

与此同时,由于噪声下降幅度更大,因此所有目标离子的SNR均得到了明显提升(6.0~11.8倍)。使用上述配置了GLS的IC系统对实验室自来水中的Cl^-^和S${O_{4}}^{2-}$ 进行测定,结果显示二者的含量分别为(0.929±0.042) mmol/L和(0.528±0.015) mmol/L。

#### 2.2.2 GLS应用于氢氧根淋洗液的IC系统

目前,IC系统所使用的氢氧根淋洗液大多通过淋洗液发生器在线制备。在该过程中,淋洗液发生器内置的杂质捕获器有效去除了杂质,因此溶液中的碳酸根含量极低,这也导致GLS在氢氧根淋洗液体系中的应用相对有限。然而,在消除样品中CO_2_的干扰方面,GLS能发挥一定作用。目前,多数氢氧根选择性色谱柱对二价离子的选择性不够好,如C${O_{3}}^{2-}$ 、S${O_{4}}^{2-}$ 与TCA之间的分离度不够理想,在测定痕量阴离子时容易受到样品中高浓度C${O_{3}}^{2-}$ 的干扰,导致无法准确定量。为此,实验分别考察了GLS使用前后对消毒副产物(TCA、DCA、Cl${O_{3}}^{-}$ )和常见阴离子(F^-^、Cl^-^、N${O_{2}}^{-}$ 、Br^-^、N${O_{3}}^{-}$ 、C${O_{3}}^{2-}$ 、S${O_{4}}^{2-}$ )分离效果的影响。如图5a所示,GLS的使用不仅降低了背景电导,还使C${O_{3}}^{2-}$ 的峰面积下降了约80%,有效改善了C${O_{3}}^{2-}$ 与TCA之间的分离效果,有利于TCA的定量分析。与此同时,在使用GLS后,约有95.7%的C${O_{3}}^{2-}$ 被脱除,有效降低了C${O_{3}}^{2-}$ 对S${O_{4}}^{2-}$ 分离的影响,从而有利于S${O_{4}}^{2-}$ 的定量分析(图5b)。

**图5:**
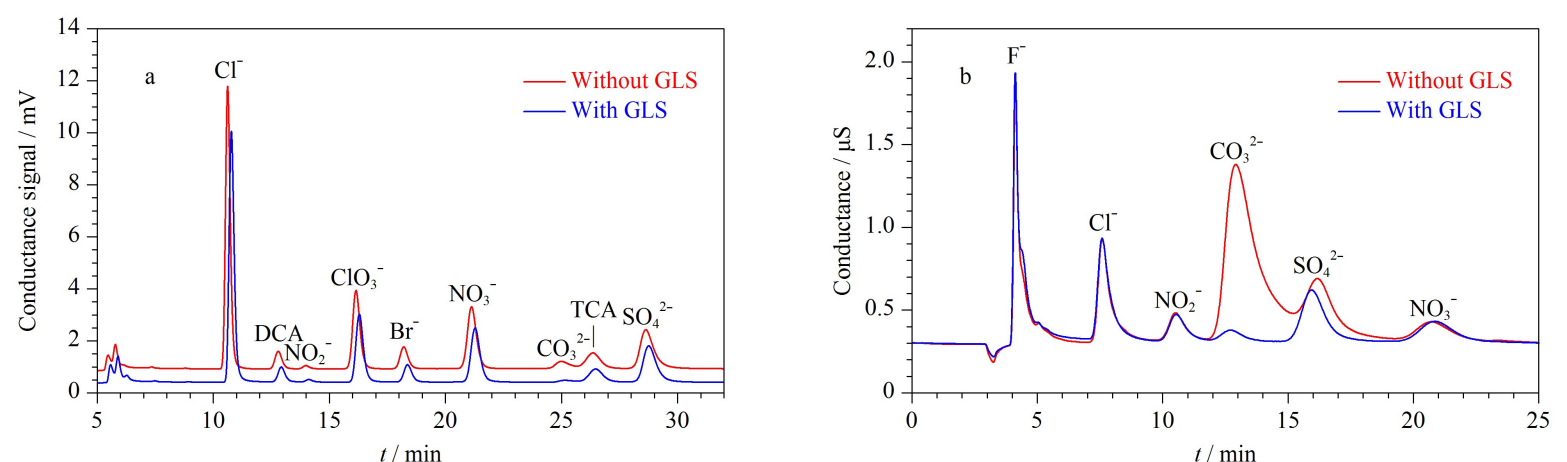
GLS应用于氢氧根淋洗液体系的效果

## 3 结论

本文设计了一种用于脱除IC系统淋洗液中CO_2_的GLS。通过将PP-T插入至内径适配的PTFE-T中形成双套管结构,将40 mmol/L的KOH溶液作为吸收液,在淋洗液流速为1 mL/min、操作温度为40 ℃的情况下,CO_2_的脱除效率可达98%以上。实验结果表明,无论是在碳酸根或氢氧根淋洗液体系中,GLS均可以有效降低系统水负峰、背景电导信号和基线噪声。同时,该GLS还可以用于大体积进样时的前处理,实现痕量阴离子的准确测定。通过更换合适的吸收液,该GLS还有望应用于铵根离子等挥发性离子的脱除,目前这些应用正处于开发阶段。
